# Decreased MIP-3α Production from Antigen-Activated PBMCs in Symptomatic HIV-Infected Subjects

**DOI:** 10.3390/pathogens11010007

**Published:** 2021-12-22

**Authors:** Fuchun Zhang, Lingling Sun, Mark K. Lafferty, Joseph B. Margolick, Alfredo Garzino-Demo

**Affiliations:** 1 Laboratory of Virus-Host Interactions, Division of Virology, Pathogenesis, and Cancer, Institute of Human Virology, Department of Microbiology and Immunology, University of Maryland School of Medicine, 725 West Lombard Street, Baltimore, MD 21201, USA; zfc8y@yahoo.com.cn (F.Z.); lsun@ihv.umaryland.edu (L.S.); mark.lafferty@umaryland.edu (M.K.L.); 2 Department of Infectious Diseases, Guangzhou No. 8 People’s Hospital, Guangzhou Medical College, Guangzhou 510060, China; 3 Department of Molecular Microbiology and Immunology, The Johns Hopkins Bloomberg School of Public Health, 615 North Wolfe Street, Baltimore, MD 21205, USA; jmargol1@jhu.edu; 4 Department of Molecular Medicine, University of Padova, 35121 Padova, Italy

**Keywords:** CCR6, MIP-3α, immune response, antigen stimulation, Th17

## Abstract

CD4^+^ CCR6^+^ T cells are highly susceptible to HIV infection, and a high cytokine producing CCR6^+^ T cell subset is selectively lost during HIV infection. The CCR6 chemokine MIP-3α (CCL20) is produced at sites of infection in SIV animal models. Recently, we have shown that MIP-3α inhibits HIV replication. This inhibition of HIV infection is mediated by CCR6 signaling and eventuates in increased APOBEC3G expression. Since there are few existing reports on the role of MIP-3α in health or disease, we studied its production by PBMCs from HIV-seronegative and HIV+ subjects. We evaluated the ability of PBMCs to produce MIP-3α in response to antigen stimulation using cells obtained from two groups: one composed of HIV-seronegative subjects (n = 16) and the other composed of HIV+ subjects (n = 58), some asymptomatic and some with clinically defined AIDS. Antigens included fragment C of the tetanus toxin, *Candida albicans*, whole-inactivated HIV, and HIV p24. MIP-3α was detected by ELISA in tissue culture supernatants of antigen-stimulated PBMCs. MIP-3α production by antigen-stimulated PBMCs was readily measured for HIV-negative subjects and for HIV-seropositive asymptomatic subjects, but not for patients with AIDS. These results suggest that subversion of the MIP-3α-CCR6 axis by HIV during the course of infection contributes to the loss of immune function that eventually leads to AIDS.

## 1. Introduction

Chemokine receptors play an important role in HIV immunopathogenesis and immune antiviral response. [1,2]. We demonstrated that CCR5 ligands are potent and specific inhibitors of certain isolates of HIV-1 [3]. Subsequently, we [4,5] and others [6,7,8,9,10,11,12,13] showed that high levels of chemokine production by antigen-activated cells are associated with protection from HIV-1 infection in high-risk subjects. More recently, studies on HIV and SIV immunopathogenesis have highlighted the role played by the MIP-3α-CCR6 axis [14,15,16,17,18,19,20,21,22,23,24,25,26].

We reported that CCR6 mediates the HIV inhibitory activity of its ligands, MIP-3α and hBD2 [26]. CCR6 is expressed on immature dendritic cells [27,28,29,30,31,32], which are among the first cells to come in contact with HIV [33,34,35,36]. Further, CCR6 is expressed on activated and memory CD4^+^ T cells, especially on lymphocytes that infiltrate inflamed mucosa [37]. Studies of CD4^+^ CCR6^+^ and CCR6^−^ cells have shown that the former are highly susceptible to HIV infection, so that protection of CCR6^+^ cells has become a goal for new approaches to HIV prevention and therapy [18,19,20,21,23,24,26,38,39,40,41,42]. Although CCR6 is at best a very marginal HIV coreceptor, it is expressed on CD4^+^ T cells that express HIV coreceptors CCR5 and CXCR4 [43,44]. CCR6 is expressed on subsets of both central and effector memory T cells that secrete high levels of IL-2 and TNF-α in response to polyclonal and antigen-specific stimulation [45]. Notably, it has been shown that CCR6^+^ T lymphocytes are lost from the peripheral blood of HIV-infected patients as disease progresses [45]. Furthermore, the capacity of CD4^+^ CCR6^+^ cells to secrete multiple cytokines remained intact among HIV-infected long-term non-progressors but was partially lost from subjects with progressive disease [45]. In the same study, CCR6^+^ T cells accumulated in the spleen of HIV-infected patients, then died by apoptosis. Perhaps as a consequence of migration, production of MIP-3α from splenocytes of HIV-infected individuals was increased, compared with HIV-seronegative controls [45]. In animal models, the expression of both MIP-3α and CCR6 are increased in acutely infected macaques and decreased in macaques with AIDS [15,46].

CCR6 is also expressed on all IL-17 producing (Th17) T cells [47,48,49]. CCR6^+^ Th17 cells are depleted in HIV-infected individuals and in pathogenic SIV rhesus macaque models of infection [19,22,38,41,50,51]. Further, rhesus macaques with larger pre-existing Th17 compartments have lower viral loads [52]. However, in SIV sooty mangabey non-pathogenic models of infection, Th17 cells are depleted during the acute phase of infection but are subsequently restored, suggesting that sustained loss of Th17 cells plays a role in disease progression [22]. In humans, the presence of mucosal Th17 cells inversely correlates with viremia, [53] and restoration of gut CD4^+^ T cells is associated with accumulation of Th17 cells [51]. Further, Th17 cells are preserved in long-term non-progressor and elite controller subjects, but not in progressor subjects [54,55,56,57].

We have shown that MIP-3α possesses antiviral activity when added post-infection and that the CCR6 ligands MIP-3α and human β-defensin 2 (hBD2) induce the expression of the host restriction factor APOBEC3G in both unstimulated and PHA-stimulated PBMCs and CD4^+^ T cells [26]. Consequently, production of CCR6 ligands could contribute to protection of CD4^+^ CCR6^+^ cells. We therefore investigated whether the CCR6 ligand MIP-3α is produced by PBMCs in response to recall antigen stimulation in HIV-negative subjects using fragment C of the tetanus toxin (TTC) and *C. albicans*. To investigate the relevance of MIP-3α in HIV infection, we measured its production in response to HIV antigens and to *C. albicans* by PBMCs from HIV-infected subjects. We detected MIP-3α production in PBMCs after antigen stimulation and found that its production was significantly greater in PBMCs of healthy subjects than in PBMCs of subjects with AIDS. Our data suggest that the CCR6-MIP-3α axis is negatively affected in the course of HIV infection.

## 2. Results

### 2.1. Antigen Stimulation-Induced Expression of MIP-3α Is Elevated in PBMCs

**Figure 1:**
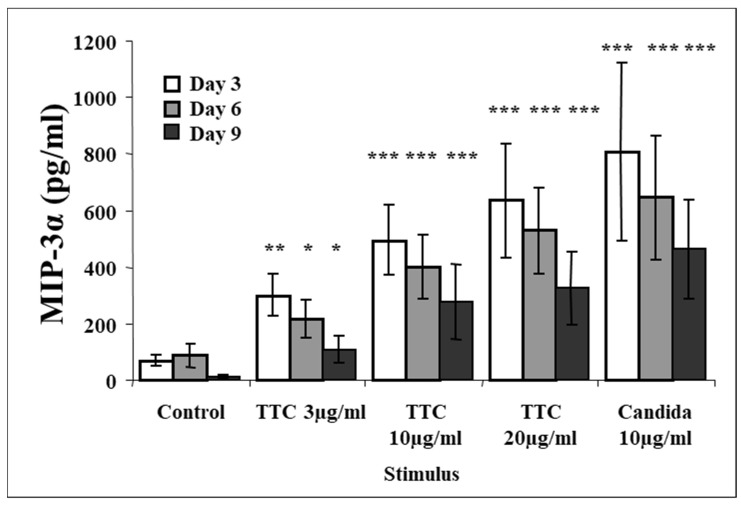
Production of MIP-3α following antigen stimulation of PBMCs at day 3, day 6, and day 9 with TTC or *Candida albicans*. PBMCs (1 × 10^6^ for each activation group) were obtained from healthy subjects and stimulated with antigens as described in the Methods section. MIP-3α was quantified by ELISA. Data are presented as mean ± SEM (n = 12). Antigen-stimulated levels significantly different from media control value at * *p* < 0.05, ** *p* ≤ 0.01, *** *p* ≤ 0.005.

Data previously published by our group showed that antigen stimulation with either TTC or *C. albicans* induced production of CCR5 chemokines [58]. Since CCR5 and CCR6 are often coexpressed in CD4^+^ T memory cells [59], we investigated whether MIP-3α is also produced upon antigen stimulation. We analyzed tissue culture supernatants from fresh PBMCs obtained from healthy volunteers (who reported having been vaccinated against tetanus) and cultured them in media without stimulation or activated with TTC or *C. albicans* or PHA. Our results show that as little as 3 µg/mL of TTC induced significant MIP-3α production as early as 3 days post-stimulation (Figure 1). At day 3, low levels of MIP-3α (69 ± 23 pg/mL, mean ± SEM) were detected in control cultures, while significantly higher levels (301 ± 79 pg/mL; *p* = 0.018) were detected in cells treated with 3 µg/mL of TTC. This effect was dose-dependent, with maximal accumulation of MIP-3α occurring in PBMCs treated with TTC at 20 µg/mL, where production was induced approximately nine-fold over control levels (induced level 633 ± 213 pg/mL, *p* = 0.011). MIP-3α production in supernatants from cells stimulated with *C. albicans* was even higher (807 ± 329 pg/mL, *p* = 0.03) than that measured in TTC-stimulated cells at any concentration, perhaps reflecting a higher number of cells that responded to this antigen or, alternatively, different potencies of each antigen. PHA was also a potent inducer of production of MIP-3α, which reached a concentration of 1426 ± 257 pg/mL (*p* = 0.0002) at day 3 after stimulation and decreased at days 6 and 9 (not shown). Production of MIP-3α was highest at day 3 for all antigens, decreasing at days 6 and 9 (Figure 1).

### 2.2. Kinetics of Release of MIP-3α

**Figure 2:**
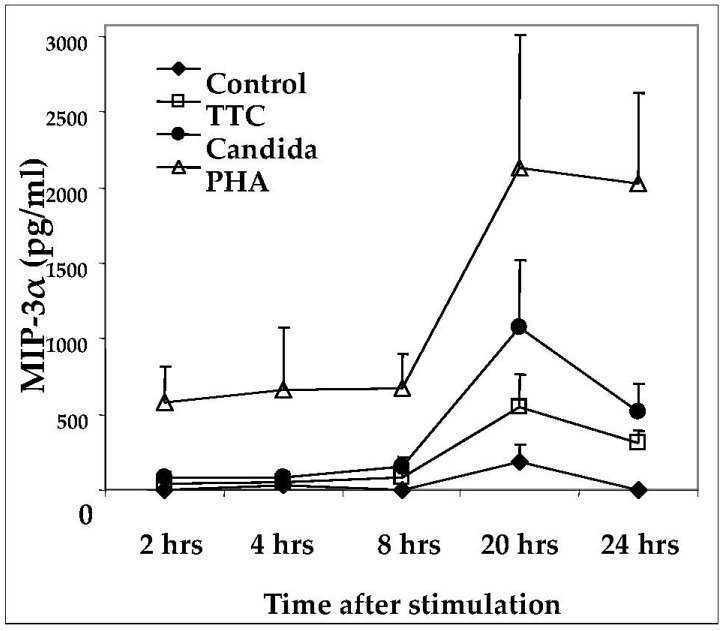
Time-course of MIP-3α production in response to antigen activation. PBMCs (1 × 10^6^ for each activation group) were obtained from healthy subjects and stimulated with antigens indicated as described in Methods. MIP-3α was quantified by ELISA. Data are presented as mean ± SEM (n = 4).

As a result of the decline in production of MIP-3α after day 3, we further evaluated the kinetics of its release post-stimulation in shorter time-course experiments. Initial experiments revealed that this chemokine is released as early as one day after activation (not shown). Next, we analyzed supernatants of PBMCs after 2, 4, 8, 20, and 24 h of antigen stimulation. MIP-3α was detectable above background levels as early as 8 h after stimulation with antigens and as early as 2 h post stimulation with PHA (Figure 2). Peak expression of MIP-3α occurred at 20 h after stimulation (Figure 2). These results suggest that chemokine release occurs relatively rapidly as a consequence of stimulation by recall antigens. 

### 2.3. MIP-3α Release as a Function of Time from Vaccination

**Figure 3:**
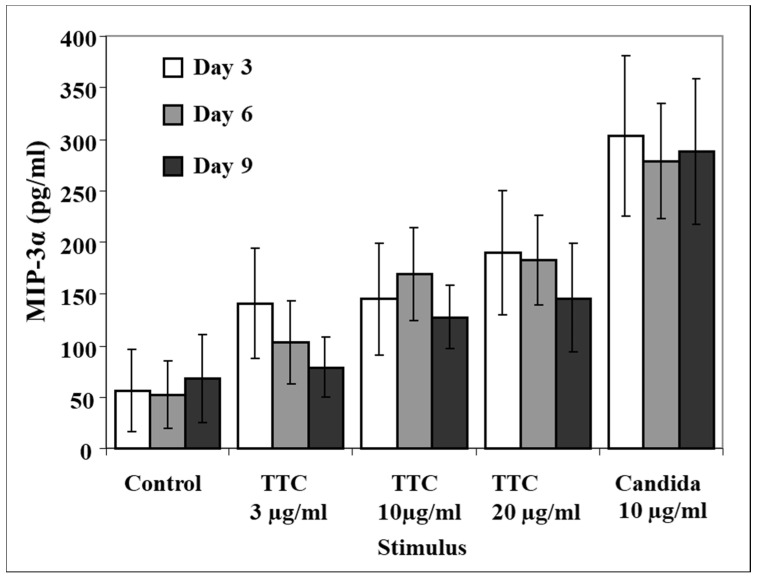
Production of MIP-3α at day 3, day 6, and day 9 following antigen stimulation of PBMCs from subjects vaccinated more than 10 years prior to testing. PBMCs (1 × 10^6^ for each activation group) were obtained from healthy subjects and stimulated with TTC or *C. albicans* as described in Methods. Production of MIP-3α was measured by ELISA. Data are presented as mean ± SEM (n = 4).

To address the question of whether the MIP-3α response to recall antigens would diminish in magnitude with time from vaccination, we analyzed MIP-3α production at 3, 6, and 9 days after stimulation using antigens in four subjects who had not received a tetanus booster at least 10 years prior to the blood donation. All of these subjects responded to activation by producing MIP-3α, but the average increase in MIP-3α induced by TTC was not statistically significant compared to control levels (Figure 3). 

### 2.4. Antigen-Induced CCR6 Ligand Release in Cryopreserved Samples

Since freshly obtained samples are not always available for study, it was important to investigate whether our assay was effective using cryopreserved specimens. An aliquot of the same PBMCs from healthy donors (n = 12) that were used to obtain the data shown in Figure 1 was cryopreserved in liquid nitrogen for four months. The cells were then thawed and stimulated using the same protocol represented in Figure 1.

**Figure 4:**
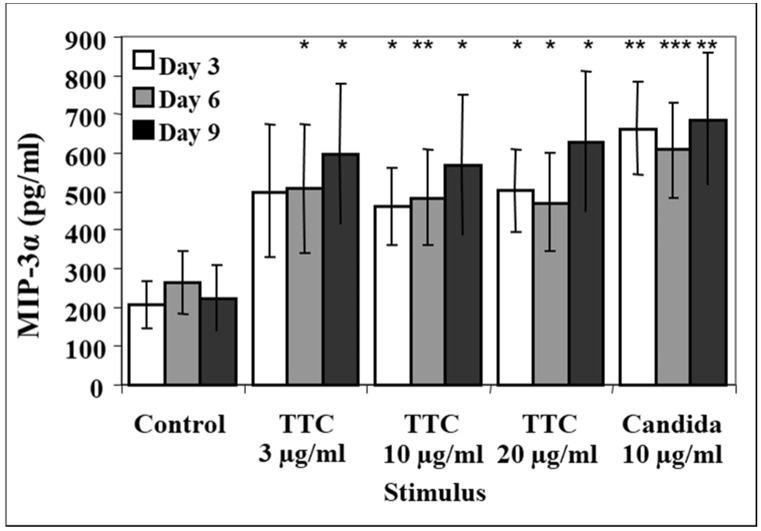
Release of MIP-3α from cryopreserved cells. PBMCs (1 × 10^6^ for each activation group) were obtained from 12 healthy subjects. Aliquots of the cells used in Figure 1 were cryopreserved in liquid nitrogen for 4 months, then thawed and stimulated. Cryopreserved cells were stimulated with 3, 10 or 20 µg of TTC or 10 µg/mL *C. albicans* or culture with media only as a control. Aliquots of supernatants were harvested at days 3, 6 and 9. MIP-3α was quantified by ELISA. Data are presented as mean ± SEM (n = 12). Antigen-stimulated values are significantly different from media control values at * *p* < 0.05, ** *p* ≤ 0.01, *** *p* ≤ 0.005.

The supernatants were harvested at days 3, 6, and 9. At day 3, the levels of MIP-3α in all antigen-stimulated groups were higher than in the control group (i.e., 207 ± 60 pg/mL; Figure 4), particularly the production in response to TTC 10 µg/mL (462 ± 99 pg/mL, *p* = 0.011) and TTC 20 µg/mL (502 ± 107 pg/mL, *p* = 0.017; Figure 4). Significantly higher levels of MIP-3α were also observed in the *C. albicans* (Figure 4) and PHA groups (not shown) than in the TTC groups. Unlike the fresh PBMCs, the cryopreserved PBMCs did not exhibit a decline in MIP-3α levels at days 6 and 9. In summary, antigen-induced levels of MIP-3α are detectable also in cryopreserved samples.

### 2.5. Antigen-Induced MIP-3α Production in PBMCs of HIV-Infected and AIDS Patients

**Figure 5:**
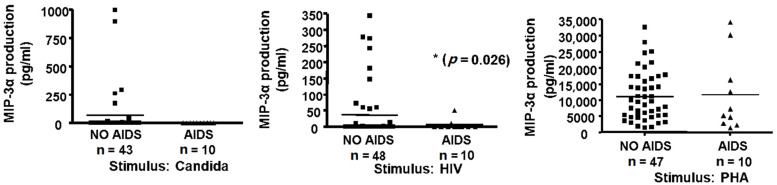
Antigen-induced MIP-3α production and AIDS status. Fresh PBMCs (1.6 × 10^6^) were cultured for 3 days as described in Methods in medium alone or with 10 µg/mL *C. albicans* (*Candida*, left panel) or with 10 µg/mL gp120-depleted, inactivated HIV-1 (HIV, middle panel), or 5 µg/mL PHA (right panel). Supernatants were collected, frozen, stored for nine years, and assayed for MIP-3α by ELISA. Control (medium alone) values were subtracted from antigen-activated values to correct for background (horizontal bars represent mean values).

Since MIP-3α production was associated with the memory response to recall antigens (Figure 1, Figure 2, Figure 3 and Figure 4) that is progressively lost in the course of HIV infection, and since we have shown that MIP-3α has HIV-inhibitory activity [26], we evaluated the production of this chemokine in supernatants from antigen-activated PBMCs that we had previously prepared in the course of a cross-sectional study of an HIV cohort [4]. In that study, cells had been stimulated for 3 days with HIV or purified p24 protein, *C. albicans*, PHA, or media alone [4]. MIP-3α levels were quantified by ELISA, and values measured in media from the stimulated group were adjusted by subtracting the background production measured with control media. We analyzed supernatants from 58 HIV-seropositive subjects, 10 of whom had progressed to AIDS and 48 of whom were asymptomatic. MIP-3α production in response to *C. albicans* did not differ significantly between these groups (Figure 5, top panel), and the same was true for PHA (Figure 5, lower panel) and p24 (not shown). However, the response to HIV was significantly lower in the male subjects with AIDS than in the asymptomatic male subjects (Figure 5, middle panel). Our data therefore suggest that the loss of immune function over the course of HIV infection includes a decreased ability to produce MIP-3α in response to the pathogen.

## 3. Discussion

In this study, we evaluated the expression of MIP-3α in the context of the immune response to protein antigens and inactivated pathogens, including TTC, *C. albicans*, HIV, and HIV p24, in both HIV-seronegative and HIV-positive subjects. MIP-3α displays expression patterns characteristic of both constitutive and inflammatory chemokines, and its production is inducible in different cell types, including many types of leukocytes [27,28,31,44,59,60,61,62,63,64,65,66,67,68]. 

Our results show that MIP-3α release was induced by antigen stimulation of PBMC. MIP-3α release was dose- and antigen-dependent, and the maximal response was to TTC 20 µg/mL group at day 3, followed by a decline at days 6 and 9. These findings are consistent with our previous findings on the release of CCR5 ligands in the course of the immune response to the same antigens used here [58].

In four subjects who had not received a tetanus booster in the past 10 years, we did not observe high enough levels of MIP-3α in response to TTC stimulation to reach statistical significance. This finding differs from our observations on CCR5 ligands MIP-1α and MIP-1ß, which were significantly induced by TTC treatment in the same samples [58]. Some caution in interpreting these data is necessary, however, since these data were obtained from a small number of subjects.

Our kinetics data showed that the production of MIP-3α occurred relatively soon after antigen stimulation, i.e., beginning at about 8 h and peaking at about 20 h. However, this is still slower than the release of CCR5 ligands, which we have shown are detectable by ELISA within 2 h of activation [58]. 

This study is the first to analyze the expression of MIP-3α protein in response to antigen stimulation in PBMCs obtained from HIV-positive subjects. One limitation of our study was that we did not identify the cells that produced MIP-3α by flow cytometry, leaving unanswered the question of which cells produced the chemokine in the conditions we tested. We observed variability in the response to all antigens, which is to be expected in a human HIV study, and probably reflects differences in immune status between patients. Our data indicate that antigen-induced production of MIP-3α is decreased in response to HIV antigens in HIV+ men with AIDS relative to HIV+ men without AIDS, suggesting that cells producing this chemokine, or their ability to produce it, are lost as disease progresses. 

Our data shows that production of MIP-3α in response to HIV is more informative than in response to *C. albicans*, p24, or PHA. The HIV+ men with AIDS in our study were viremic, and an association between viremia and decreased ability to respond to HIV antigens has been reported [69]). Our data differ from data on MIP-3α levels in serum and plasma, which previous reports showed to be consistently higher than those measured in HIV-seronegative donors [70,71]. This discrepancy may be due to MIP-3α levels in plasma and serum likely being the result of chemokine production by a variety of sources, including neutrophils, and it is not clear whether these high levels would be relevant in the tissues, where HIV replication and immune response are critical. Indeed, levels of MIP-3α were found to be lower in the small intestine mucosa of HIV-infected individuals as compared to HIV- seronegative controls, a finding that correlated with impaired restoration of Th17 following antiretroviral therapy. Further, a study of expression of cytokines from ectocervix samples in the context of HIV infection found lower levels of production of MIP-3α in HIV-infected women as compared to HIV-seronegative controls [72]. In addition, a study of mucosal tissues from HIV-2-infected individuals suggested that levels of MIP-3α in the lamina propria correlated with markers of mucosal integrity [73]. Thus, the decrease in production of MIP-3α by antigen-activated cells in HIV-infected subjects with AIDS may contribute to a reduced mucosal integrity and ability to control HIV in vivo. In one study, a CD90^+^ subpopulation of cells producing high levels of MIP-3α was selectively depleted in the course of HIV infection, providing a potential explanation for our findings [41]. Decreased production of MIP-3α in response to antigen activation is also likely to reflect the loss of HIV-specific cells observed in the course of HIV infection, so that CCR6^+^ cell numbers and MIP-3α production in response to HIV antigens may be useful as markers of disease progression [74]. Alternatively, it is possible that the loss of MIP-3α production might impede interactions between MIP-3α-secreting cells and CCR6-expressing cells, which include DC and B and T cells, and thus might contribute to the loss of cellular immunity observed in the course of HIV infection. 

## 4. Materials and Methods

### 4.1. Study Subjects

We used samples (supernatants from PBMCs) that we had obtained from two previous studies:

(a) A study of antigen stimulation on PBMCs from healthy donors. PBMCs from 16 healthy (by self-assessment), HIV-negative donors were obtained by venipuncture and Histopaque isolation as described [59]. Of these, 4 donors reported not having been vaccinated against tetanus for more than 10 years, while the other subjects had been vaccinated within the previous 10 years. All subjects signed informed consent forms approved by the Institutional Review Board.
(b) Studies on subjects from an HIV cohort. Subjects were obtained from the Baltimore–Washington DC center of the Multicenter AIDS Cohort Study (MACS), a longitudinal study of the natural history of HIV-1 infection in men who have sex with men [4]. Briefly, 1253 men were recruited in 1984–85 and in 1987–90 and followed at 6-month intervals with clinical and laboratory testing as well as storage of repository specimens. For this study, blood was obtained from 58 HIV-1 seropositive men at visit 27 (April–October 1997). HIV-positive subjects were categorized into two groups: 10 with AIDS and 48 who were asymptomatic and did not have AIDS according to the 1993 definition by the Centers for Disease Control and Prevention. Thirteen control, non-MACS HIV-seronegative subjects with no history of exposure to HIV were recruited from the laboratory staff at the Institute of Human Virology.

### 4.2. Cells and Laboratory Studies

(a) HIV-seronegative subjects. PBMCs were incubated with 3–20 µg/mL Fragment C of tetanus toxin (TTC–Calbiochem, La Jolla, CA, USA), 10 µg/mL *Candida albicans* (Greer Laboratories, Lenoir, NC, USA), or 2.5 µg/mL phytohemoagglutinin (PHA; Sigma-Aldrich, St Louis, MO, USA). Supernatants from cells incubated with media alone were used as controls. Supernatants were collected on days 3, 6, and 9 after activation and frozen at −80 °C. Supernatants had been stored for about 4 years prior to testing for MIP-3α.
(b) PBMC obtained from HIV-1 seropositive subjects in the MACS were collected in CPT tubes (Falcon-BD, Franklin Lakes, NJ, USA), following the instructions of the manufacturer. Fresh PBMCs were cultured in round-bottom 96-well plates (Falcon-BD) in RPMI medium (GIBCO-Invitrogen, Carlsbad, CA, USA) supplemented with 10% human AB serum and antibiotics (100 U/mL penicillin, 100 U/mL streptomycin) (GIBCO-Invitrogen) at a density of 2 × 105 cells/100 µL. Cells were incubated for 3 days with 10 µg/mL gp120-depleted, inactivated HIV-1, 10 µg/mL purified p24 antigen (see below), 10 µg/mL *C. albicans*, or 2.5 µg/mL PHA, or with media alone as a control. Supernatants were collected on day 3 (for PHA) or day 6 (for antigen stimulation) and frozen at −80 °C [4]. HIV-1 HZ321 immunogen was obtained by concentration and purification from the supernatant fluid of HZ321-infected HUT-78 cells. In the preparation of the immunogen, envelope gp120 was depleted during freezing and thawing and during the purification process [75].

### 4.3. MIP-3α Measurement

Human MIP-3α was measured using commercial ELISA plates pre-coated with an anti-hMIP-3α monoclonal antibody from R&D Systems (Minneapolis, MN, USA), following the manufacturer’s instructions. 

### 4.4. Statistical Analysis

Data of patients were abstracted from clinical records and maintained in an Excel database. Analysis of data distribution and of statistical significance was performed with GraphPad InStat, Version 4.00, for Windows XP (GraphPad Software, San Diego, CA, USA) using a two-tailed, parametric (paired *t*-test, for normally-distributed data) or non-parametric (Wilcoxon signed rank test or Mann–Whitney test for non-normally distributed data) statistical tests. The level of statistical significance attained is indicated in the text and in figure legends. Intervention studies involving animals or humans, and other studies that require ethical approval, must list the authority that provided approval and the corresponding ethical approval code.

## Data Availability

The data presented in this study are available on request from the corresponding author.
